# Supplementary material for: Classical criticality via quantum annealing
Source: Nat Commun. 2026 Jan 15;17:856. doi: 10.1038/s41467-025-67568-w (PMC12827436; doi:10.1038/s41467-025-67568-w)
Supplement: Supplementary file 1 — Supplementary Information [file 41467_2025_67568_MOESM1_ESM.pdf]

Supplemental Material for "Classical Criticality via Quantum Annealing"

Pratik Sathe,[1,2,*] Andrew D. King,[3] Susan M. Mniszewski,[4]
Carleton Coffrin,[5] Cristiano Nisoli,[1,†] and Francesco Caravelli[1,6,‡]

[1] *Theoretical Division, Quantum & Condensed Matter Physics, Los Alamos National Laboratory*
[2] *Information Science & Technology Institute, Los Alamos National Laboratory, Los Alamos, NM 87545, USA*
[3] *D-Wave Quantum Inc., Burnaby BCV5G 4M9, Canada*
[4] *Computing and Artificial Intelligence (CAI) Division (CAI-3 Information Sciences), Los Alamos National Laboratory*
[5] *Advanced Network Science Initiative, Los Alamos National Laboratory, Los Alamos, NM 87545, USA*
[6] *Dipartimento di Fisica dell'Università di Pisa, Largo Bruno Pontecorvo 3, I-56127 Pisa, Italy*

I. EXACT SOLUTION AND CRITICAL LINE EXPRESSIONS OF THE PUD MODEL

The study of exactly solvable models in statistical mechanics began with Onsager's landmark solution of the ferromagnetic Ising model on a two-dimensional square lattice [1]. Later, Villain introduced a distinct exactly solvable model—the so-called "odd model" [2], now more commonly referred to as the fully frustrated Ising model—where frustration is introduced systematically across the lattice. Building on these foundational works, Ref. [3] provided the first exact solution of a model that interpolates between Onsager's and Villain's limits, which they referred to as the "Piled-Up Dominoes Model" (PUD).

Using dimer methods, the model has also been solved more recently in Ref. [4]. Following the Montroll-Potts-Ward method, the partition function per site was obtained to be

$$\begin{aligned} \frac{\log Z}{N} &= \log(2\cosh(2J/\kappa T)) \\ &+ \frac{1}{4\pi^2}\int_0^\pi d\phi \int_0^\pi d\theta \, \log\left[(1+\tilde{z}^2)^2 - 2\tilde{z}^2(\cos\phi + \cos\theta)\right], \end{aligned} \tag{1}$$

where $\tilde{z} = \tanh(2J/\kappa T)$, and κ is Boltzmann's constant [4]. Locations where Z is not analytic can be found, yielding two critical lines: (i) for $s \leq 1$, a line separating the ferromagnetic (FM) phase from the paramagnetic (PM) phase, and (ii) for $s \geq 1$, a line separating the antiferromagnetic (AFM) phase from the PM phase. The FM-PM critical line was found to be given by

$$s(T) = \frac{1}{2}\left(T \tanh^{-1}\left(\frac{z^3 + z^2 + z - 1}{z^3 + z^2 - z + 1}\right) + 1\right), \tag{2}$$

where $z = \tanh\left(\frac{J}{\kappa T}\right)$. At $s = 0$, the critical temperature is Onsager's critical temperature T_c.

An alternative, equivalent form of the model's critical line can be extracted analytically by identifying parameter values at which long-range order vanishes. This method is applicable to both FM-PM and AFM-PM critical lines. Defining $J'/J = 1 - 2s$, $K = \beta J$ and $K' = \beta J'$ with $\beta = 1/(k_B T)$, the two critical lines are given by [3]:

$$\sinh(2K)\sinh(K + K') = 1, \tag{3a}$$

$$\text{and } \sinh(2K)\sinh(K + K') = -1. \tag{3b}$$

Substituting $K' = (1 - 2s)K$, we find

$$\sinh(2K)\sinh(2K(1 - s)) = \pm 1. \tag{4}$$

Solving for s, we obtain the two critical lines:

$$s_\pm(T) = 1 - \frac{T}{2}\sinh^{-1}\left(\frac{\pm 1}{\sinh(2/T)}\right), \tag{5}$$

where the '+' sign corresponds to the FM-PM critical line, and the '−' sign corresponds to the AFM-PM phase transition.

---

* Current Address: D-Wave Quantum Inc., Burnaby BCV5G 4M9, Canada
psathe@dwavesys.com
† nisoli@lanl.gov
‡ francesco.caravelli@proton.me

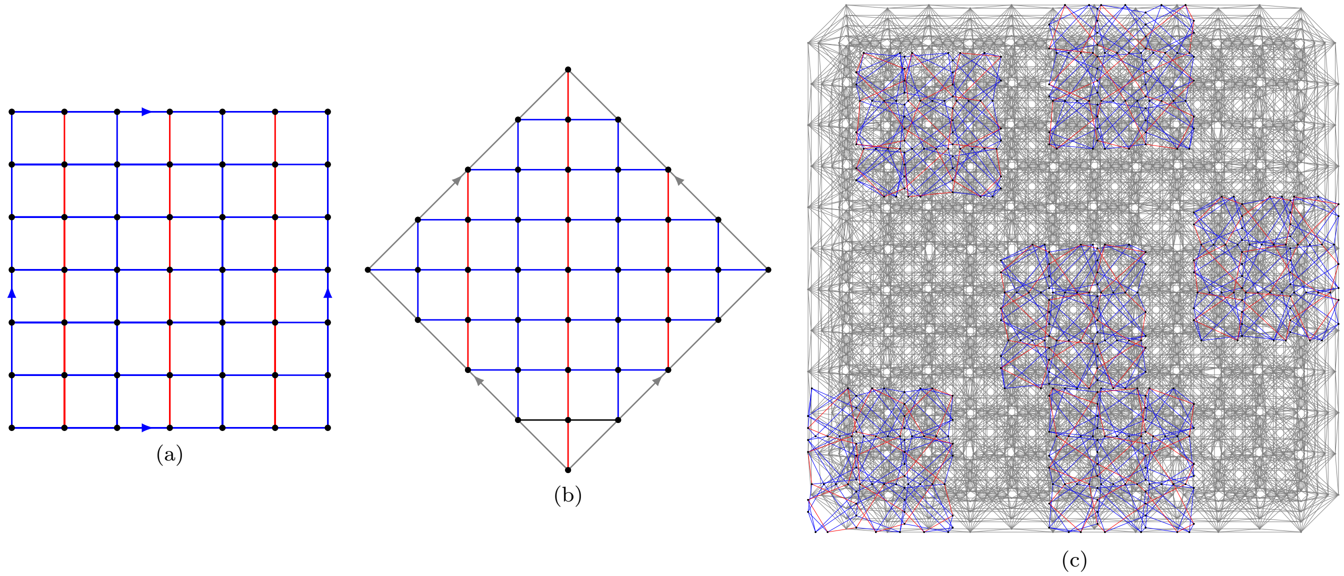

Supplementary Figure 1. The connectivity graphs for (a) normal and (b) skew toroidal systems for two example system sizes. Each spin is shown by a black dot, and red and blue connections correspond to couplings with $J = 1 - 2s$ and $J = s$ values respectively. (a) Normal toroidal system corresponding to $L = 6$ and number of spins $N = L^2 = 36$. The spins in the top line are identified with the spins in the bottom line, and similarly, the vertical edges on the left and on the right are identified with each other. (b) The connectivity for the skew toroidal system corresponding to $L = 8$ with $N = L^2/2 = 32$ number of spins. The spins on the slanting lines (in gray) are identified (with the orientations specified by the arrows). (c) The 6 disjoint embeddings for the sampling experiments corresponding to system size $N = 72$ (skew torus with $L = 12$). The gray dots and gray lines denote the nodes and edges of the Zephyr graph connectivity of the `Advantage2_prototype2.6` device used for the experiments. The red ($J = 1 - 2s$) and blue ($J = 1$) couplers are superimposed on top of the Zephyr graph.

II. EMBEDDINGS

Graphical representations of the systems corresponding to $N = 36$ and $N = 32$ are shown in Figs. 1a and Fig. 1b respectively.

To implement sampling experiments for any system size, the first step is to map—or embed—the toroidal system of interest onto the physical qubits available on the QPU. This mapping must preserve the required connectivity, ensuring that all interacting spins are appropriately connected via couplers. However, the connectivity graph of the problem is often different from that of the QPU hardware. In such cases, it may not be possible to assign each logical spin directly to a physical qubit, and one must instead use chains—groups of connected physical qubits that represent a single logical spin. To minimize errors due to broken chains, we avoid chains altogether in our experiments (by using a chain length of 1 for each qubit), ensuring that each logical spin is mapped to exactly one physical qubit.

Furthermore, to obtain more samples per experiment, for each value of N, we used multiple disjoint embeddings on the Zephyr graph connectivity of the `Advantage2_prototype2.6` device. After each anneal, the measurement outputs were processed to extract the corresponding number of spin configurations. We generated these embeddings for the QPU graph using the `find_subgraph` function in the `minorminer.subgraph` module, applied to different, disjoint regions or subgraphs of the QPU graph. For example, we generated six disjoint embeddings that were then used for the $N = 72$ sampling experiments (see Fig. 1c).

The various system sizes used and the number of corresponding embeddings are listed in Table II.

We note that for the PUD model, most couplers are ferromagnetic for any value of s. (In fact, for $s < 0.5$, all the couplers are ferromagnetic.) We found that the quality of the samples was adversely affected by cross-talk and memory effects when most couplers are ferromagnetic, and required a longer pause between consecutive anneals for better result quality. To reduce these errors, we implemented a gauge transformation $\sigma_{x,y} \to (-1)^{x+y}\sigma_{x,y}$, which flips the values of all alternate spins. This results in the sign of each coupler being flipped, $J_{x,y} \to -J_{x,y}$. Consequently, the model transforms to one in which all horizontal and alternate vertical columns have antiferromagnetic bonds, while all the remaining alternate vertical columns are ferromagnetic, resulting in most bonds being antiferromagnetic. We obtain the samples generated using QA for the gauge-transformed Hamiltonian, undo the gauge transformation by flipping each measured spin configuration, and then compute the ferromagnetic and antiferromagnetic order parameters.

Number of spins (N)	Type(Normal/Skew)	L	Number of embeddings
32	Skew	8	22
36	Normal	6	16
64	Normal	8	6
72	Skew	12	6
100	Normal	10	2
128	Skew	16	2
144	Normal	12	2

Supplementary Table I. Various system sizes used for the finite-size scaling analysis

We note that random gauge transformations have been used in prior works, for example, in Refs. [5–8], to reduce the effects of non-idealities in the hardware. Unlike these methods, we use a fixed gauge transformation described above, and work with it throughout, since having a fixed gauge also enables a simpler calibration refinement procedure which is described below.

III. CALIBRATION REFINEMENT AND EXPERIMENTAL PARAMETERS

Here, we describe the calibration refinement techniques used for our experiments.

The importance of the calibration refinement procedure is evident when comparing various physical quantities and observables, such as the Binder cumulant, heat capacity and magnetization susceptibility, obtained from quantum annealing without this refinement. In Fig. 2, we plot these quantities with the same annealing parameters as Fig. 2 in the main text, but without calibration refinement. In addition to the prominent increase in the noise in the data, we find no peaks in the χ/β and C_V/β^2 plots, even after implementing experiments at very low energy scales ($J^{-1} \approx 25$). While peaks might appear at even lower energy scales, the associated measurements become increasingly noisy due to the finite precision with which such small energy differences can be implemented. Additionally, these peaks (if observed at all), would indicate a critical energy scale that departs significantly from values expected from the approximate transition region seen in the order parameter plot (Fig. 1c in the main text). In the same vein, we conclude that the crossing of the Binder cumulant curves seen in Fig. 2a, is inaccurate.

Let us briefly outline the main ideas underlying shimming before providing more details of the procedure. While sampling from an Ising model that has some symmetries (such as a $\mathbb{Z}_2$ symmetry, or more general graphical symmetries), certain statistical quantities such as mean magnetization of individual spins and the so-called frustration probability of couplers (defined below) are expected to satisfy corresponding conditions in an ideal implementation. During our calibration refinement procedure, we adjust the values of the FBOs (flux-bias offsets) at each individual qubit and coupler strengths (individual values of $J_{i,j}$) by small amounts, to nudge the system to satisfy these symmetries.

The two main diagnostic quantities that we used for the calibration refinement protocol are average magnetization for each spin, m_i, and coupler frustration probability f_{ij} for the coupler that connects qubits i and j. These quantities are defined as [9]

$$m_i = \langle s_i \rangle, \tag{6a}$$

$$\text{and } f_{ij} = \frac{1 + \langle s_i s_j \rangle \operatorname{sign} J_{ij}}{2}, \tag{6b}$$

with $\langle . \rangle$ denoting the average of a quantity of 100 measurements. At any value of s, the PUD model has an external longitudinal field value of 0 at each spin. Consequently, on average, we expect the magnetization m_i (without taking the absolute value) of each spin to be 0. Using gradient descent (with step size denoted by α_Φ), we adjust the values of FBOs for each qubit individually, to lead toward an average magnetization of 0.

On the other hand, we do not have a quantitative prediction of the value of f_{ij} for any coupler. Nonetheless, at any value of $s \neq 0$ for a toroidal system, there are three “coupler orbits” [9]. Referring to Fig. 1a, these orbits form 3 sets:

1. Orbit 1: All horizontal couplers.

2. Orbit 2: All vertical couplers belonging to the blue columns.

3. Orbit 3: All vertical couplers belonging to the red columns.

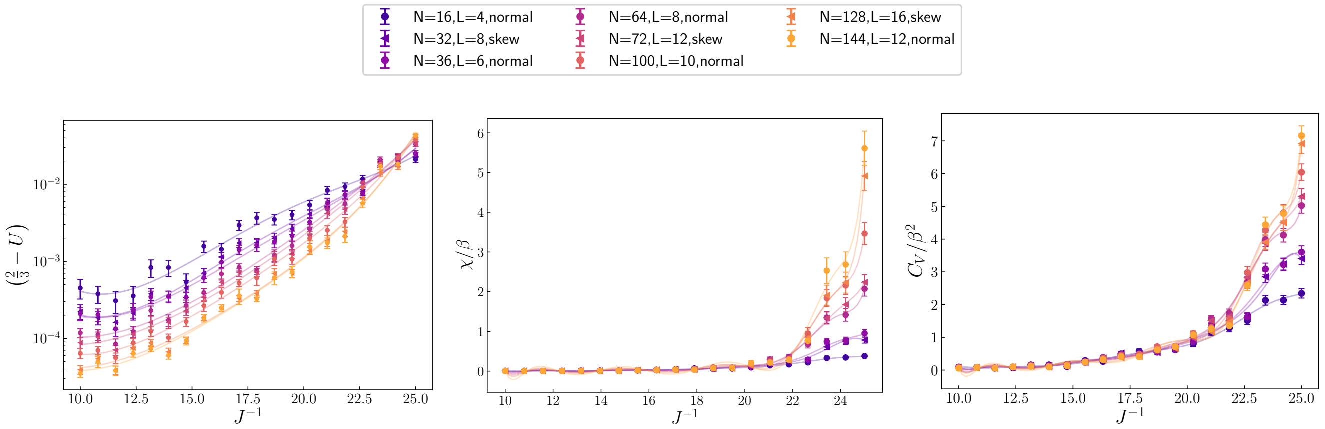

(a) (b) (c)

Supplementary Figure 2. (a) The Binder cumulant, (b) magnetization susceptibility times temperature and (c) heat capacity times temperature squared, obtained without implementing the calibration refinement procedure deviates significantly from those obtained with it (cf Fig. 2 in the main text). Shown here are the results for $s = 0$ with annealing parameters identical to those in Fig. 2 from the main text. The solid lines are fifth order polynomial fits.

On a toroidal geometry, all couplers within any orbit should have the same frustration probability. We use gradient descent (with step size denoted by α_J) and adjust the value of each coupler strength to lead the system towards smaller spreads in frustration probability within each coupler orbit.

We implemented calibration refinement separately for each value of $s \in \{0, 0.2, 0.4, 0.6, 0.8\}$ and a grid of values of J^{-1}. We further incorporated smoothing of FBOs and coupler strengths across energy scales at each iteration, and an adaptive adjustment of gradient descent step sizes (α_Φ and α_J) [9]. The full calibration refinement procedure for a fixed value of s is provided in Procedure 1.

We used $N_{\text{shim}} = 1000$ shimming iterations, and initial gradient descent step sizes $\alpha_J = 0.02 \times J$, and $\alpha_\Phi = 10^{-6}$. We implemented a smoothing of the FBOs and coupler values across the various energy scales starting at iteration number 75. The adaptive step size procedure was also implemented from iteration 75, with a look-back of 20 iterations to determine whether to increase or decrease the step sizes by a factor of 0.1. Shimming of coupler values was started at iteration 50, while the FBO shimming was started at step 20.

The values of coupler strengths and FBOs for all the system sizes and the energy scale grid for each s were then used to obtain $10,000$ spin configurations. Our estimates and corresponding standard deviation values for the Binder cumulant, and temperature-scaled magnetization susceptibility χ/β and heat capacity C_V/β^2 were computed using 1,000 bootstrap resamples with 1,000 samples each.

For all sets of values (N_{spins}, J, s) which were studied for the finite size scaling analysis, we observed the desired trends: a decrease in the spread of the magnetization values as well as of the frustration probability values after implementing the calibration refinement procedure. As an example, we show the histograms of the two quantities before (i.e., at iteration 0), and after (i.e., at the last iteration) for $N = 16$, $s = 0$ and an energy scale value located approximately at the center of the grid of values.

Lastly, we turn to the effect of the number of samples generated from quantum annealing on the accuracy of our results. We recall that 10,000 spin configurations for every combination (s, J, N) were obtained for the reported results. To emulate the effects of increasing the number of samples, we implement bootstrap experiments with 1000 resamples, with each bootstrap set comprising of increasing sizes, ranging from 20 to 2000 configurations. We find that the Binder cumulant, χ/β and C_V/β^2 values consistently stabilize at around 500 configurations, indicating that increasing the number of configurations is unlikely to change the results.

A. Finite Size Scaling using temperature-scaled magnetization susceptibility

The locations (i.e., temperature values) and heights of the peaks of $\chi(L, T)$ for various L can be used to determine ν, γ and T_c using a finite size scaling (FSS) analysis. We claimed in the main text that for large system sizes, it is justifiable to apply identical FSS procedures to the peaks of χ/β instead. Here, we provide an argument in support of this claim. (We have dropped the dependence of s, since it plays no role below.)

Procedure 1: Calibration refinement at a fixed s for extracting χ/β, C_V/β^2, and Binder cumulant

Input : (i) For a value of interest of s, a grid of N_{grid} values of J^{-1} centered at the anticipated value of the critical energy scale. (ii) A list of values of system sizes N. (iii) For each N, an embedding with $\mathcal{M}_{\text{embd}}(N)$ number of disjoint embeddings that maps the model of size N to the QPU graph.

Procedure: For each of system size N, implement the following steps:

1. Implement the gauge transformation $s_{x,y} \to (-1)^{x+y} s_{x,y}$ for each of the $\mathcal{M}_{\text{embd}}(N)$ number of disjoint embeddings. All embeddings combined, let us denote the qubits by q_i, the FBOs by Φ_i with $i \in \{1, \ldots, N \times \mathcal{M}_{\text{embd}}\}$.
2. Divide the list of couplers into three sets corresponding to the three coupler orbits specified above.
3. Choose initial gradient descent step sizes α_Φ and α_J. Initialize FBO values $\Phi_i = 0$ for all i.
4. For each value of energy scale J in the chosen grid of values, initialize the coupler strengths to be $J_{i,j} = J \times (1 - 2s)$ or $J_{i,j} = J \times 1$ chosen appropriately.
5. For each value of energy scale J in the chosen grid of energy scale values, implement the following steps.
   (a) Using current values $\{J_{ij}\}$ and $\{\Phi_i\}$ generate 100 samples from quantum annealing. Obtain the average magnetization m_i for each used qubit i, and frustration probability f_{ij} for each used coupler ij, averaged over the measurements. (See Eqs. 6.)
   (b) For each qubit i, set
   $$\Phi_i \leftarrow \Phi_i - \alpha_\Phi m_i. \tag{7}$$
   (c) For each of the three coupler orbits, compute average frustration probabilities within the orbit. For each used coupler i, j, update
   $$J_{ij} \leftarrow J_{i,j}(1 + \alpha_J(f_{ij} - \overline{f})), \tag{8}$$
   where $\overline{f}$ denotes the average frustration probability of the coupler orbit that coupler (i, j) belongs to.
   (d) Update the values of α_J and α_Φ using the history of values of frustration probabilities and average qubit magnetizations using the adaptive step size procedure outlined in Ref. [9].
6. For each qubit i, implement a moving window (of size 3) average for Φ_i, across the N_{grid} number of inverse energy scale values. Obtain updated values of Φ_is for each energy scale.
7. Similarly, for each coupler (i, j), implement a moving window (of size 3) average of J_{ij} across the N_{grid} number of inverse energy scale values. Obtain updated values of J_{ij}s for each energy scale.
8. Repeat steps 5-7 N_{shim} number of times.

Output: Coupler and FBO values for all the qubits used for sampling, for all inverse energy scale grid values.

We start with the finite-size scaling ansatz

$$\chi(L, t) = L^{\gamma/\nu} f(L^{1/\nu} t), \tag{9}$$

where $t = (T - T_c)/L$ denotes the reduced temperature.

We note that the location of the peak of χ/β for a system size L, denoted by $\widetilde{T}_L$ here, satisfy

$$0 = \left.\frac{\partial(\chi/\beta)}{\partial T}\right|_{\widetilde{T}_L} \tag{10}$$

$$= \chi(L, \widetilde{t}_L) + (1 + \widetilde{t}_L) \left.\frac{\mathrm{d}\chi(L, t)}{\mathrm{d}t}\right|_{\widetilde{T}_L}. \tag{11}$$

The location of the peak of χ for system size L, denoted here by T_L, satisfies

$$\left.\frac{\mathrm{d}\chi(L, t)}{\mathrm{d}t}\right|_{T_L} = 0, \tag{12}$$

$$\implies f'(L^{1/\nu} t_L) = 0. \tag{13}$$

From Eq. (11) and Eq. (9), we get

$$(1 + \widetilde{t}_L) = -\frac{f(L^{1/\nu}\widetilde{t}_L)}{f'(L^{1/\nu}\widetilde{t}_L) L^{1/\nu}} \tag{14}$$

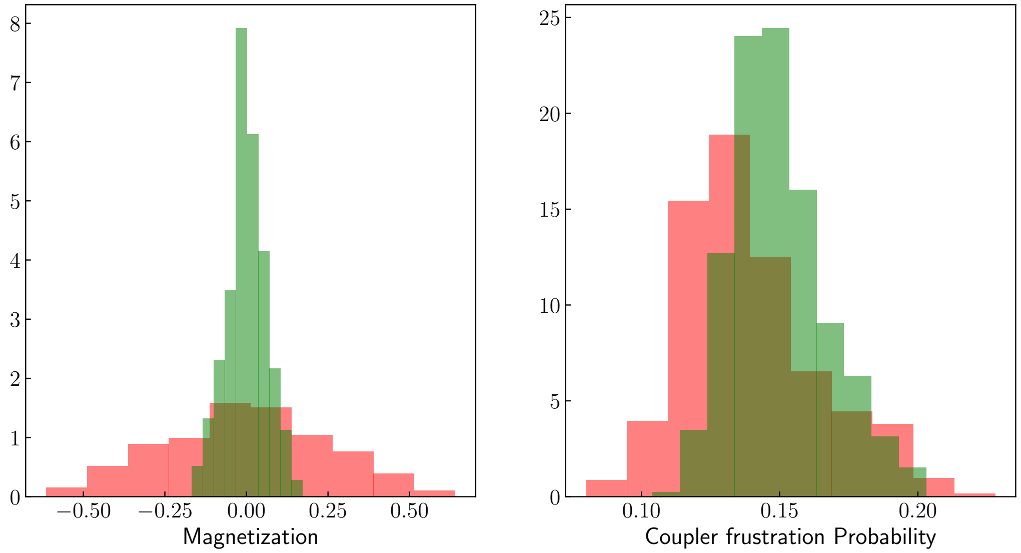

Supplementary Figure 3. Improvements in the individual qubit magnetization (left) and coupler frustration probabilities (right) after the calibration refinement procedure. The histograms before the refinement procedure (red) are noticeably more spread out, than at the end of the refinement procedure (green). Shown here are the statistics for $s = 0.0$, $N = 16$ and $J = 0.0547$, which lies roughly at the center of energy scale grid that was chosen for the finite size scaling analysis.

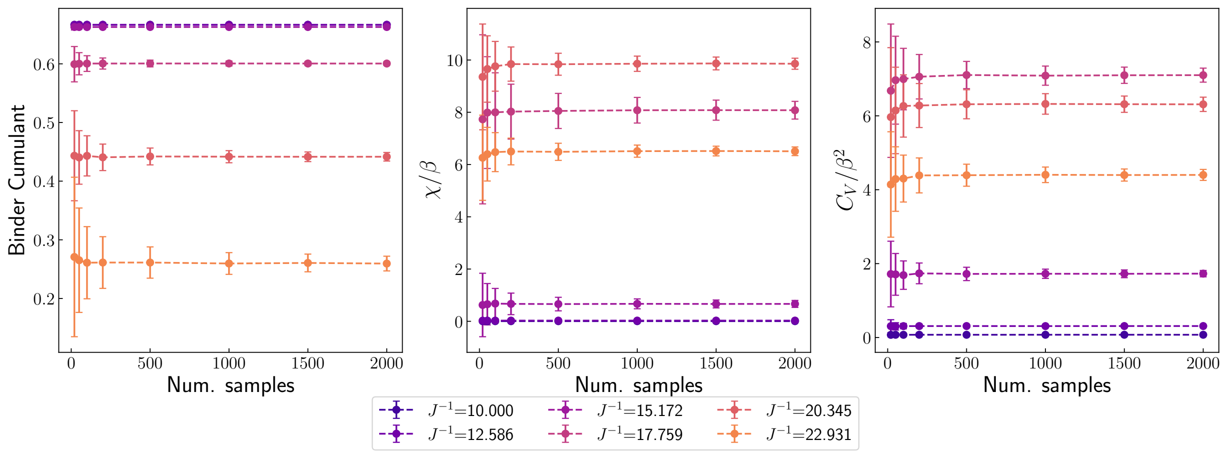

Supplementary Figure 4. Increasing the number of quantum annealing samples (i.e., spin configurations) does not significantly change the values of the Binder cumulant, χ/β and C_V/β^2 beyond approximately 500 samples. To simulate this, we implement bootstrap resampling with increasing sample sizes, from 10,000 spin configurations obtained from QA for each experiment. Shown here is the evolution of these three quantities with increasing sample size for the 12×12 toroidal system. Values at all energy scales we consider converge (even though only five energy scale values are plotted to avoid cluttering the plots). The error bars indicate one standard deviation about the mean.

Taylor expanding the numerator as well as the denominator in $\Delta_L t := \widetilde{t}_L - t_L$, we get

$$(1+\widetilde{t}_L) = -\frac{f(L^{1/\nu}t_L) + f''(L^{1/\nu}t)|_{t_L} L^{2/\nu}(\Delta_L t)^2 + O(\Delta_L t^3)}{L^{1/\nu}\Delta_L t f''(L^{1/\nu}t)|_{t_L} + O(\Delta_L t^2)} \frac{1}{L^{1/\nu}} \tag{15}$$

Here, w used Eq. (13) to eliminate the first order derivative of f. For large L, the dominant terms in the numerator and the denominator are the respective first terms. Therefore, we get

$$(1+\widetilde{t}_L) \approx -\frac{1}{L^{2/\nu}} \frac{f(L^{1/\nu}t_L)}{\Delta_L t f''(L^{1/\nu}t)|_{t_L}} \tag{16}$$

$$\text{or } \Delta_L t \approx -\frac{1}{L^{2/\nu}} \frac{1}{1+\widetilde{t}_L} g(L^{1/\nu}t_L), \tag{17}$$

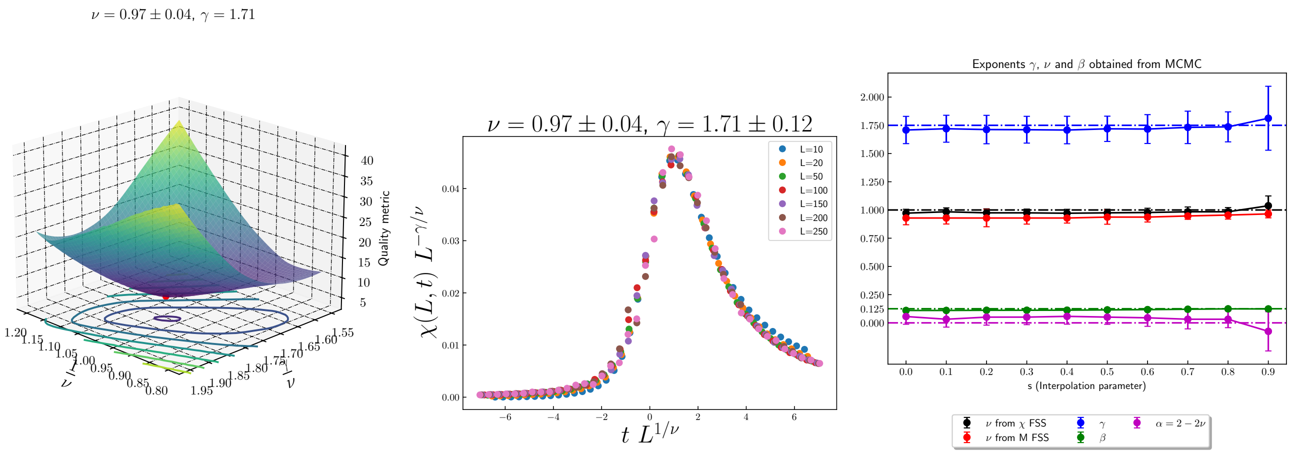

(a) (b) (c)

Supplementary Figure 5. Markov-chain Monte Carlo results. (a) The quality metric, Eq. (23) as a function of $\frac{1}{\nu}$ and $\frac{\gamma}{\nu}$ is minimized at $\frac{1}{\nu} = 1.2$ and $\frac{\gamma}{\nu} = 1.95$ for $s = 0$. (b) The corresponding data collapse for χ at $s = 0.0$. (c) Extracted values of the critical exponents as a function of s obtained using finite size scaling (FSS). The dashed lines indicate the known exact values at $s = 0.0$.

where $g(x) = \frac{f(x)}{f''(x)}$. We note that $g(L^{1/\nu}t_L)$ is a constant independent of L, since it equals the value of $f(x)/f''(x)$ at a fixed x (specifically, the x corresponding to the maximum of $f(x)$). Since we also expect $\widetilde{t}_L \approx 0$ for large L, we can also ignore the $(1 + \widetilde{t}_L)$ term in the denominator. Hence, we arrive at the expression

$$\Delta_L t \sim -\frac{1}{L^{2/\nu}}, \tag{18}$$

which approaches 0 as $L \to \infty$.

In other words, the location of the peak of χ/β as a function of T is a good approximation for the location of the peak of χ as a function of T for large system sizes.

Let us now compare the heights of the peaks for the two cases. Their ratio equals

$$\frac{(\chi/\beta)|_{\text{peak}}}{\chi|_{\text{peak}}} = \frac{T_c(1 + \widetilde{t}_L)\chi(\widetilde{t}_L, L)}{\chi(t_L, L)} \tag{19}$$

To first order in $\Delta_L t$, $\chi(\widetilde{t}_L, L) = \chi(t_L, L)$ due to Eq. (13). Hence, conclude that

$$\frac{(\chi/\beta)|_{\text{peak}}}{\chi|_{\text{peak}}} \approx T_c(1 + \widetilde{t}_L) \tag{20}$$

$$\approx T_c, \tag{21}$$

where we used $(1 + \widetilde{t}_L) \approx 1$ for large L. Hence, for large system sizes, we expect that the peak heights of χ/β to be the same as the peak heights of χ, but scaled by a constant factor of T_c.

IV. MONTE CARLO SIMULATIONS

We recall that the values of the exponents for the $s = 0$ limit of the PUD model (i.e., the ferromagnetic Ising model on the 2d square lattice) are known from the exact solution. Although the expression for the partition function of the PUD model (in the absence of a longitudinal field) has been obtained via exact solutions for all values of s, the critical exponents are unknown for $s \neq 0$, to our knowledge.

In the main text, we probed the critical exponents γ and ν for the ferromagnetic-paramagnetic phase transitions for $s \in [0, 1)$. To verify the correctness of the QA results, we compute the exponents γ, ν and β using the Metropolis-Hastings Markov-chain Monte Carlo (MCMC) algorithm, and infer the exponent α using the hyperscaling relation $d\nu = 2 - \beta$.

We recall that the exponents are defined via the relations $\xi \propto t^{-\gamma}$, $\chi \propto t^{-\nu}$, $C_V \propto t^{-\alpha}$ and $m \propto t^{\beta}$ as $t \to 0$, where t denotes the reduced temperature.

We implemented single-flip MCMC simulations for $s = 0, 0.1, \ldots, 0.9$ at intervals of 0.1. For each value of s, we implemented simulations for toroidal systems of size $L \times L$ with L values $10, 20, 50, 100, 150, 200, 250$. For each simulation, we initialized the system in a random spin configuration partially polarized (at 70%) along the $+z$ direction. (This was done to avoid getting stuck in the so-called 'striped' metastable states, which are known to occur if a random zero magnetization configuration is used as the initial configuration [10].) Next, we implemented 40,000 steps per spin to let the system equilibrate.

To obtain estimates of errors in our estimates of various thermodynamic quantities, we first computed the autocorrelation function $\chi(t)$ for each simulation [11]. The autocorrelation time was obtained using the formula $\tau = \sum_{t=0}^{T} \frac{\chi(t)}{\chi(0)}$. Standard deviation errors for average magnetization and average energy were then computed using the equation

$$\sigma = \frac{2\tau}{t_{\max}} \left(\overline{m^2} - \overline{m}^2 \right). \tag{22}$$

Using the autocorrelation time τ, the number of statistically independent samples is estimated to be $\mathcal{N}/2\tau$, where $\mathcal{N}$ denotes the number of Monte Carlo steps per spin for each simulation. For all our MCMC simulations, we implemented $\mathcal{N} = 2,000,000$ steps per spin. To obtain standard deviation errors for the fourth-order Binder cumulant, magnetization susceptibility χ, and heat capacity C_V, we used 1000 bootstrap datasets, with each dataset containing $\mathcal{N}/2\tau$ number of samples.

To obtain critical exponents, we used the data collapse method. Specifically, we obtained estimates of γ and ν, we plotted $\chi(L,T)L^{-\gamma/\nu}$ vs $tL^{1/\nu}$, where $t = (T - T_c)/T_c$ for various values of γ and ν. The values resulting in the best data collapse were obtained using a modified version of the quality metric described in Ref. [12]. Specifically, we used the quality metric

$$P = \frac{1}{\mathcal{N}} \sum_{p} L_p^{\gamma/\nu} \sum_{j \neq p} \sum_{i,\text{over}} \left| L_j^{-\gamma/\nu} \chi(L_j, t) - g_p(L^{1/\nu} t) \right|. \tag{23}$$

Here, we introduced the normalization factor $L_p^{\gamma/\nu}$ to ensure that indefinitely increasing γ does not spuriously result in a smaller quality factor. The critical exponents and along with their errors, were then obtained by minimizing the quality metric using the Nelder-Mead method. As a representative example, we show a 3d visualization of the quality metric for the data collapse for χ at $s = 0$ in Fig. 5a, and the corresponding data collapse in Fig. 5b.

A similar data collapse analysis was implemented for average magnetization, in order to obtain the exponents ν and β. To extract the exponent α, we used the hyper-scaling relation $d\nu = 2 - \beta$.

A plot of the extracted exponents as a function of s is shown in Fig. 5c.

V. BINDER CUMULANT CURVES FOR SMALL SYSTEM SIZES

In the thermodynamic limit, Binder cumulant curves corresponding to different system sizes are expected to intersect at a single, fixed temperature—the critical temperature. For relatively small system sizes, however, a scatter in the intersection points is expected due to finite-size effects [13], as also documented in previous studies [14, 15]. We observe such scatter in our quantum annealing results. As a consistency check, we confirm that similar behavior occurs in classical Monte Carlo simulations (see Fig. 6a), in addition to a slight deviation from the exact critical temperature.

VI. CRITICAL SLOWING DOWN

In the main text, we showed that the normalized autocorrelation function, $\chi(t)/\chi(0)$ for the QA samples does not exhibit an exponential drop with increasing t, which is typically observed in MCMC simulations. Instead, at any value of energy scale, the QA data exhibits a roughly constant value of the correlation time for $t > 1$. Critical slowing down can be characterized by the corresponding time scale (called the autocorrelation time) diverging with increasing system size and decreasing distance from the critical temperature. As an alternative metric, we instead compute the average value of the normalized autocorrelation function over $t \in \{1, \ldots, 100\}$ for the largest system size. Across all values of s examined, this average remains low and shows no noticeable peak near the extracted inverse critical temperature (see Fig. 6b)—once again indicating the absence of critical slowing down in QA sampling.

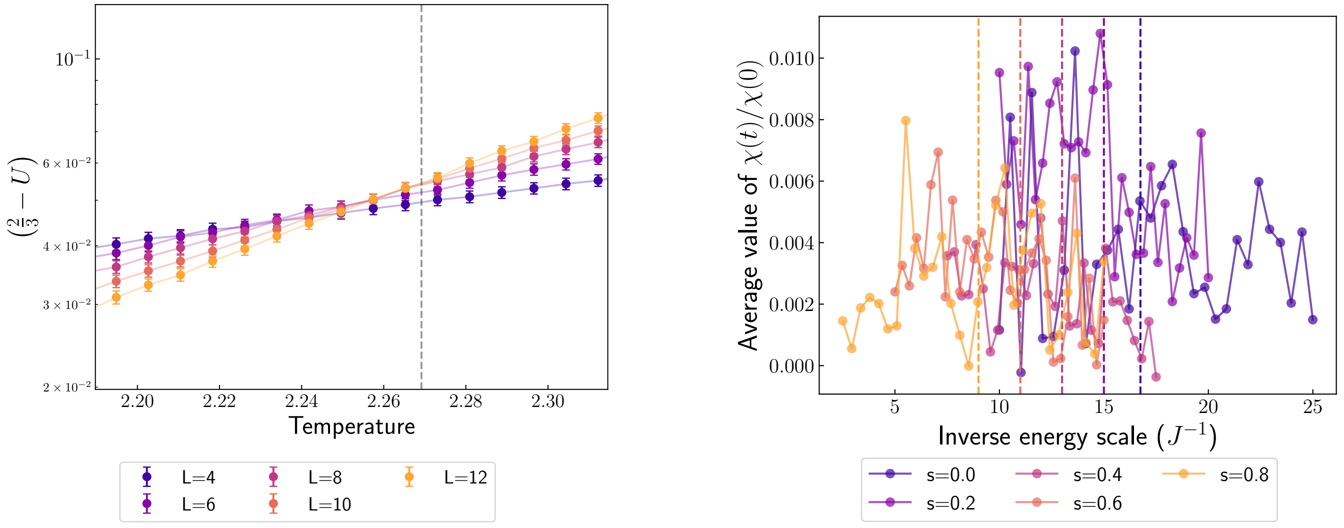

(a) (b)

Supplementary Figure 6. (a) A scatter in the intersection of the Binder cumulant curves is obtained for $L \times L$ toroidal systems (of similar sizes as the ones implemented in the quantum annealing experiments) at $s = 0$. The critical temperature from the exact solution is shown as a vertical line for reference. (b) The average value (over 100 steps) of the normalized autocorrelation function, $\chi(t)/\chi(0)$, for quantum annealing samples for various values of s, as a function of the inverse energy scale. Vertical lines mark the extracted values of critical inverse energy scale.

[1] L. Onsager, Crystal Statistics. I. A Two-Dimensional Model with an Order-Disorder Transition, Physical Review **65**, 117 (1944).

[2] J. Villain, Spin glass with non-random interactions, Journal of Physics C: Solid State Physics **10**, 1717 (1977), `https://dx.doi.org/10.1088/0022-3719/10/10/014`.

[3] G. André, R. Bidaux, J.-P. Carton, R. Conte, and L. de Seze, Frustration in periodic systems : Exact results for some 2D Ising models, Journal de Physique **40**, 479 (1979), `http://dx.doi.org/10.1051/jphys:01979004005047900`.

[4] F. Caravelli, Some exactly solvable and tunable frustrated spin models, Physica A: Statistical Mechanics and its Applications **594**, 127007 (2022), `https://linkinghub.elsevier.com/retrieve/pii/S0378437122000863`.

[5] S. Mandrà, Z. Zhu, and H. G. Katzgraber, Exponentially Biased Ground-State Sampling of Quantum Annealing Machines with Transverse-Field Driving Hamiltonians, Physical Review Letters **118**, 070502 (2017), `https://link.aps.org/doi/10.1103/PhysRevLett.118.070502`.

[6] R. Sandt and R. Spatschek, Efficient low temperature Monte Carlo sampling using quantum annealing, Scientific Reports **13**, 6754 (2023), `https://www.nature.com/articles/s41598-023-33828-2`.

[7] J. Nelson, M. Vuffray, A. Y. Lokhov, T. Albash, and C. Coffrin, High-Quality Thermal Gibbs Sampling with Quantum Annealing Hardware, Physical Review Applied **17**, 044046 (2022), `https://link.aps.org/doi/10.1103/PhysRevApplied.17.044046`.

[8] J. Marshall, D. Venturelli, I. Hen, and E. G. Rieffel, Power of Pausing: Advancing Understanding of Thermalization in Experimental Quantum Annealers, Physical Review Applied **11**, 044083 (2019), `https://link.aps.org/doi/10.1103/PhysRevApplied.11.044083`.

[9] K. Chern, K. Boothby, J. Raymond, P. Farré, and A. D. King, Tutorial: Calibration refinement in quantum annealing, Frontiers in Computer Science **5** (2023), `https://www.frontiersin.org/articles/10.3389/fcomp.2023.1238988`.

[10] V. Spirin, P. Krapivsky, and S. Redner, Freezing in Ising ferromagnets, Physical Review E **65**, 016119 (2001), `https://link.aps.org/doi/10.1103/PhysRevE.65.016119`.

[11] M. E. Newman and G. T. Barkema, *Monte Carlo methods in statistical physics* (Clarendon Press, 1999).

[12] S. M. Bhattacharjee and F. Seno, A measure of data collapse for scaling, Journal of Physics A: Mathematical and General **34**, 6375 (2001), `https://dx.doi.org/10.1088/0305-4470/34/33/302`.

[13] K. Binder, Finite size scaling analysis of Ising Model block distribution functions, Zeitschrift für Physik B Condensed Matter **43**, 119 (1981), `https://doi.org/10.1007/BF01293604`.

[14] K. Binder and D. W. Heermann, *Monte Carlo Simulation in Statistical Physics: An Introduction*, Graduate Texts in Physics, Vol. 0 (Springer, Berlin, Heidelberg, 2010) `https://link.springer.com/10.1007/978-3-642-03163-2`.

[15] A. M. Ferrenberg and D. P. Landau, Critical behavior of the three-dimensional Ising model: A high-resolution Monte Carlo study, Physical Review B **44**, 5081 (1991).
